# Spatial transcriptome analysis of myenteric plexus and intestinal epithelium of colon in patients with Parkinson’s disease

**DOI:** 10.1186/s40478-025-02047-3

**Published:** 2025-07-05

**Authors:** Chaewon Shin, Karoliina Eliisa Ruhno, Jung Hwan Shin, Sanha Hwang, Jasper Roldan Go, Minji Kang, Hyun Je Kim, Ji Hwan Moon, Han-Joon Kim

**Affiliations:** 1 Department of Neurology, Neuroscience Center, Seoul National University Bundang Hospital, 82, Gumi-ro 173 Beon-gil, Bundang-gu, Seongnam-Si, 13620 Republic of Korea; 2 Department of Biomedical Sciences, Seoul National University College of Medicine, 103, Daehak-ro, Jongno-gu, Seoul, 03080 Republic of Korea; 3 Department of Neurology, Seoul National University Hospital, Seoul National University College of Medicine, 101, Daehak-Ro, Jongno-Gu, Seoul, 03080 Republic of Korea; 4 Samsung Genome Institute, Samsung Medical Center, 81, Irwon-ro, Gangnam-gu, Seoul, 06351 Republic of Korea

**Keywords:** Parkinson’s disease, Gastrointestinal tract, Alpha-synuclein, Myenteric plexus, Intestinal epithelium, Spatial transcriptomics

## Abstract

Alpha-synuclein (AS) accumulation is found in the nerve plexuses of the gastrointestinal tract in patients with Parknison’s disease (PD). Moreover, alterations in microbiome composition and its metabolites were confirmed in the colon of patients with PD. However, there has been no study that evaluates transcriptomic alterations of the nerve plexus and intestinal epithelium simultaneously using in vivo tissue of patients with PD. Therefore, we aimed to investigate the gene expression profiles of the myenteric plexus and intestinal epithelium of the colon of patients with PD. Ten full-depth slides of paraffin-embedded surgical specimens of the colon or rectum from five patients with PD and five controls were included. AS accumulation was found in the myenteric plexus in all patients with PD. We performed spatial-specific transcriptomic profiling of the myenteric plexus and epithelium using the GeoMX Digital Spatial Profiler. Forty-one differentially expressed genes (DEGs) (36 up-regulated and 5 down-regulated) were identified in the myenteric plexus of patients with PD compared to controls. In the intestinal epithelium, 240 DEGs (81 up-regulated and 159 down-regulated) were identified. Pathway analysis showed upregulated response to type II interferon and lymphocyte activation, while downregulated cellular response to zinc and copper ions in the intestinal epithelium of patients with PD. In the myenteric plexus, neuroepithelial cell differentiation and axon development were upregulated. Network analysis showed the following key genes: and *HLA-DRA*, *SERPINA1*, and metallothioneins in the intestinal epithelium, and *LAMP1*, *TUBB2A*, and *S100B* in the myenteric plexus. This study suggests that inflammatory processes may occur in the intestinal epithelium, while neuronal regeneration mechanisms may be active in the myenteric plexus in patients with overtly developed PD. A spatial transcriptomic analysis of the brain and the gastrointestinal tract will enable a better understanding of the gut-brain axis in PD.

**Supplementary Information:**

The online version contains supplementary material available at 10.1186/s40478-025-02047-3.

## Introduction

Parkinson’s disease (PD) is a neurodegenerative disorder caused by the loss of dopaminergic neurons in the substantia nigra of the brain. The pathologic hallmark of PD is the accumulation of intraneuronal alpha-synuclein (AS), known as Lewy bodies or Lewy neurites, in the brain. Studies have demonstrated that AS accumulation is also observed in the gastrointestinal (GI) tract of patients with PD [1, 2]. Furthermore, AS accumulations have been identified in peripheral tissues obtained prior to the onset of PD [2], and in the GI tissues of patients with idiopathic rapid eye movement sleep behavior disorder, which is a prodromal stage of PD [3]. These findings have highlighted the GI tract as a key organ potentially involved in the pathophysiology of PD, leading to an increasing number of studies focused on the gut-brain axis in PD.

Environmental changes, including alterations of the gut microbiome and related metabolites such as short chain fatty acids, occur in the colon of patients with PD [4]. These changes contribute to the dysfunction of tight junctions in the intestinal epithelium, resulting in a “leaky gut” that allows the invasion of bacteria and environmental toxins [5]. Consequently, enteric glial cell activation and gut inflammation occur, which may be related to the AS accumulation in the enteric nervous system in PD [4]. The accumlated AS is speculated to ascend to the brain through the vagus nerve and initiate pathological changes in PD [6]. The environmental and pathologic alterations in the GI tract of patients with PD imply that there may also be changes in the transcriptome in the cells of the gut wall. However, studies that evaluate gene expression alterations in the GI tract of patients with PD are scarce. Only one study investigated gene expression alterations in the colonic epithelium of patients with PD [7]; the study analyzed rectal biopsy samples from 12 patients, examining 770 genes associated with neuropathological and neuroinflammatory pathways. They identified alterations in 22 genes related to neuroglial and mitochondrial functions, vesicular trafficking, and inflammation.

Understanding the gut-brain axis could provide deeper insights into the pathophysiology of PD and facilitate the development of innovative therapeutic strategies. Recent advancements in spatial transcriptomics have enabled precise identification of gene expression alterations across various tissue types including the GI tract. By applying this technology, we aimed to simultaneously analyze gene expression alterations in nerve cells in both the myenteric plexus and the intestinal epithelium in a single tissue slide of the colon in patients with PD. 

## Materials and methods

### Participants

We reviewed the medical records of eligible patients at the Seoul National University Hospital (SNUH) and selected those who were diagnosed with PD and underwent radical surgery in the colon or rectum for cancer treatment from 2004 to 2019. The inclusion criteria for the PD group were as follows: (1) clinical diagnosis of PD which was evaluated by experienced movement disorder specialists; (2) Diagnosis of PD secured at the medical record of a final follow-up visit more than 1 year after the onset of symptoms; (3) age at symptom onset > 50 years; (4) radically resected surgical specimen of the colon or rectum available at the pathology bank of SNUH; and (5) permission to use the specimen for research purposes. The exclusion criteria were as follows: (1) clinical diagnosis of other parkinsonian syndromes, such as Parkinson plus syndrome or secondary parkinsonism; (2) known genetic causes; (3) presence of any inflammatory disease in the surgical specimen; and (4) chemotherapy or radiotherapy prior to the surgery.

Normal controls were selected from the pathology database of SNUH. The eligibility criteria for the control group were as follows: (1) absence of parkinsonism, dementia, or other neurological diseases in the medical record, which could potentially affect the results; (2) absence of any inflammatory disease in the surgical specimen; and (3) no chemotherapy or radiotherapy prior to surgery. The study design was approved by the Institutional Review Board of SNUH (IRB No. H-2206–222-1337). A waiver of informed consent met the requirements and was granted.

### Clinical evaluation

Clinical characteristics were collected by chart review including sex, age at the time of surgery, age at symptom onset of PD, age at diagnosis of PD, and surgical site. The symptom onset of PD was defined as the onset year of any of the motor symptoms. Durations from symptom onset to surgery and to diagnosis were calculated from the age information.

### Selection of specimens

We collected one formalin-fixed paraffin-embedded (FFPE) block per patient from the proximal or distal margin of the surgical specimens, which were the most distant normal areas from the cancer lesions as observed in previous studies [3, 8]. All tissue specimens from the participants were transferred to the pathology department immediately after surgical resection and fixed within one hour. Thereafter, the samples were processed and preserved as FFPE blocks using a standardized protocol of the Pathology Department of SNUH. Five serial 4-μm slides per collected block were sectioned. First two sections were used for immunohistochemistry (IHC), next slide was used for spatial transcriptomic analysis, and the last two slides were reserve slides.

### IHC

The slides were de-waxed, rehydrated, and incubated with primary antibodies on automated machines as previously described [3, 8]. A primary antibody against phosphorylated AS (pAS) (1/1000 anti-pAS at serine 129 monoclonal Ab [EP1536Y]; Abcam ab51253, Cambridge, UK) was used along with the Leica Bond-III system, in accordance with the manufacturer’s instruction. Bound antibodies were detected using the Bond Polymer Refine Detection system (Leica Biosystems, Wetzlar, Germany). The next slide was stained with a primary antibody against neurofilament (NF) (1/2000 anti-NF monoclonal Ab; DAKO clone 2F11, Santa Clara, CA, USA) using the BenchMark XT system (Ventana Medical Systems, Inc., Oro Valley, AZ, USA).

### Pathological evaluation

All stained slides were scanned using a Leica Slide Scanner (Aperio GT 450 DX; Leica Biosystems, Nussloch, Germany). Digital slides were evaluated using a pathology slide viewing software (Aperio ImageScope ver. 12.4; Leica Biosystems, Nussloch, Germany).

The presence of AS accumulation was conservatively defined as in the previous studies [3, 9] as (1) occurrence of definite and clear positive staining patterns such as “dots and fibers” or “Lewy body-like staining” pattern on immunohistochemical staining of pAS and (2) localization in neural structures confirmed through histologic inspection and positive NF staining.

### NanoString GeoMx manual RNA FFPE slide preparation

The experiments were performed using NanoString GeoMx Digital Spatial Profiler (DSP) (NanoString Technologies, Seattle, WA, USA) according to manufacturer’s instructions (Manual Slide Preparation MAN-10150-02, NGS DSP Instrument User Manual: MAN-10116-05, Library Preparation & GeoMx NGS Pipeline: MAN-10153-03) with minor adjustments. FFPE tissue Sects. (4 µm thick) were baked at 37 °C overnight and then at 60 °C for 1 h. The sections were then deparaffinized and rehydrated by placing in CitriSolv three times, 5 min each time, followed by 100% EtOH two times, 5 min each time, 95% EtOH for 5 min, and 1X PBS wash gradient for 1 min in staining jars. Target retrieval was then performed by dipping the slides for 10 s in diethylpyrocarbonate (DEPC) water and 20 min in 1X Tris–EDTA in a steamer heated to ~ 99 °C. This was followed by a 5-min 1X PBS wash at room temperature. Next, RNA targets were exposed by placing slides in a staining jar containing 1 µg/mL of proteinase K diluted in 1X PBS and incubating them in a 37 °C water bath for 15 min, followed by a 5-min wash in 1X PBS at room temperature. After RNA target exposure, post-fix preservation was done by washing the slides in 10% neutral buffered formalin (NBF) for 5 min, then NBF stop buffer two times, 5 min each time, and 1X PBS for 5 min. Overnight hybridization was performed in a hybridization chamber at 37 °C, by covering each slide with 200 µL of a mixture of Buffer R, Whole Transcriptome Atlas RNA detection probes, and DEPC water according to the protocol. The next day, the slides were stringently washed in a 37 °C water bath two times, 25 min each time with a stringent wash solution, and were then washed with 2X saline-sodium citrate (SSC) two times, 2 min each time at room temperature. The slides were then covered with 200 µL of Buffer W for blocking in a humidity chamber at room temperature for 30 min, and then with 200 µL of morphology marker mix according to the protocol, for 1 h. The morphology markers used were SYTO 13, CD34/PECAM (4H11, Abcam, Cambridge, UK), protein gene product 9.5 (PGP 9.5) (CLN-EPR4118, Abcam, Cambridge, UK), and α-smooth muscle actin (α-SMA) (EPR5368, Abcam, Cambridge, UK). Finally, the slides were washed with 2X SSC two times, 5 min each time. Subsequently, the slides were taken to the GeoMx DSP machine.

### NanoString GeoMx DSP

Slides were loaded into the NanoString GeoMx DSP (NanoString Technologies, Seattle, WA, USA) after being covered with 6 mL of Buffer S. Per slide, 12 regions of interest (ROIs) were selected: 6 ROIs from the myenteric plexus and 6 ROIs from the intestinal endothelium. ROIs were manually drawn based on prior pAS and NF IHC staining, and guided by morphology markers CD34/PECAM, PGP 9.5, and αSMA used in GeoMx slide staining. Only ROIs with nuclei count exceeding 20 were selected. The chosen ROIs were individually illuminated using UV light, allowing the photocleavage of the oligonucleotides from the antibodies bound in the ROIs. These were then collected on a 96-well microwell, and stored at −20 °C.

### Library generation and pooling

Before library processing, the 96-well plate was thawed, spun down, and dehydrated at 65 °C for 1 h in a thermo-cycler with the lid kept open. Samples were then individually rehydrated with 10 µL of nuclease-free water and prepared for PCR by combining each sample with 2 µL of GeoMx Seq Code PCR Master Mix, 4 µL of primer, and 4 µL of DSP aspirate. After library synthesis, 4 µL of each sample were pooled together and subjected to AMPure cleanup. The pooled sequencing library was then quality controlled using Agilent TapeStation 4150 for each pooled sample group and sequenced on a NovaSeq 6000 instrument (Illumina Inc., San Deigo, CA, USA) using pared-end 2 × 27 base paired reads.

### Data analysis

The raw reads from FASTQ files were processed using NanoString’s GeoMx NGS pipeline version 2.3.3.10 and saved as Digital Count Conversion (DCC) files. Initial quality control (QC) was performed using GeoMx DSP Analysis Suite version 2.4.2.2. First, segment and biological probe QC were conducted with preset values. The data was not filtered, and all segments and targets were considered for further analysis. The data were analyzed using R (version 4.4.1) and a customized in-house pipeline, which consists of sequential steps including batch correction, normalization, and differential gene expression analysis. To reduce technical variability across samples, batch correction was performed using the betweenLaneNormalization function [10], which adjusts for global differences in overall signal intensity between ROIs. This was followed by normalization using the RUVSeq package [11], which identifies and removes unwanted sources of variation by leveraging replicate structure. Together, these preprocessing steps enhance the comparability of expression profiles across ROIs and improve the reliability of downstream differential analysis. Differential analysis was carried out on the normalized counts using DESeq2 [12] to identify differentially expressed genes (DEGs) between the two groups. DESeq2 uses Benjamini–Hochberg method to control false discovery rate (FDR) and the DEGs were selected based on adjusted p-value. To further investigate and discover the key DEGs, we performed network analysis using network propagation on BioGRID protein–protein interaction (PPI) network [13]. We mapped the genes in the data to the PPI network and initialized the nodes with the fold change values of the genes. We used random walk with restart (RWR) algorithm to rank the genes in the network. The equation applied by RWR is as follows:

$$p^{{t + 1}} = \left( {1 - r} \right)W^{\prime}p^{t} + rp^{0}$$

where ${{\varvec{p}}}^{0}$, ${{\varvec{p}}}^{t}$, and ${{\varvec{p}}}^{t+1}$ are the probability vector of the nodes initialized with the fold change, the probability vector of the current stage, and the probability vector of the next stage, respectively. ${{\varvec{W}}}{\prime}$ is column-normalized adjacency matrix representing the PPI network. $r$ is the restart rate.

We also calculated betweenness centrality for each gene on the network to quantify the topological importance of the genes. Betweenness centrality is a measure of the shortest paths of all pairs of nodes that pass through a node. The equation is shown below:

$$BC\left(v\right)=\sum_{s\ne v\ne t\in V}\frac{{\sigma }_{st}(v)}{{\sigma }_{st}}$$

where $V$ is a set of nodes, ${\sigma }_{st}$ is the total number of shortest paths from node $s$ to node $v$, and ${\sigma }_{st}(v)$ is the number of shortest paths from node $s$ to node $v$ passing through $v$. Using the two metrics, network propagation result and betweenness centrality, we were able to discover a set of key DEGs.

#### Statistical analysis

The clinical characteristics of the participants were compared using non-parametric tests such as the Mann–Whitney U test and Fisher’s exact test, owing to the small number of participants. A two-tailed *p*-value ≤ 0.05 was considered statistically significant. Statistical analyses were performed using SPSS version 26.0.0.0 (IBM Corp., Armonk, NY, USA).

#### Role of the funding source

The funder of the study had no role in the study design, data collection, data analysis, data interpretation, or writing of the manuscript. The corresponding author had full access to all data in the study and had the final responsibility for the decision to submit the manuscript for publication.

## Results

### Clinical characteristics and results of pAS IHC

The characteristics of the participants are summarized in Table 1. A total of five patients with PD and five controls were included in the study, all of whom underwent radical surgery for cancer in the colon or rectum. Percentage of female patients were not statistically different between patients with PD (40%) and controls (20%; *p* = 1.000). The mean age at surgery for patients with PD and controls was 68.2 ± 6.1 and 73.2 ± 5.5 years, respectively, with no significant difference (*p* = 0.421). The mean age at symptom onset in the PD patient group was 64.4 ± 6.4 years, and the mean duration from symptom onset to surgery was 3.8 ± 5.7 years. AS accumulation was observed in the myenteric plexus of all patients with PD, whereas all controls were negative.

**Table 1:** Clinical characteristics of participants

Item	Patients with PD					Controls					*p*-value
Sex	F	F	M	M	M	F	M	M	M	M	1.000
Age at surgery (years)	62	75	69	62	73	69	74	82	73	68	0.421
Age at onset of motor symptoms (years)	59	69	57	65	72						
Age at diagnosis (years)	59	74	57	65	73						
Duration from onset to diagnosis (years)	0	5	0	0	1						
Duration from onset to surgery (years)	3	6	12	-3	1						
Surgery	RHC	LAR	AR	RHC	RHC	LHC	RHC	RHC	RHC	AR	
pAS positivity in IHC	( +)	( +)	( +)	( +)	( +)	(–)	(–)	(–)	(–)	(–)	0.008

PD, Parkinson’s disease; pAS, phosphorylated alpha-synuclein; IHC, immunohistochemistry; RHC, right hemicolectomy; LHC, left hemicolectomy; AR, anterior resection; LAR, low anterior resection

### Results of spatial transcriptomic profiling

**Fig. 1:**
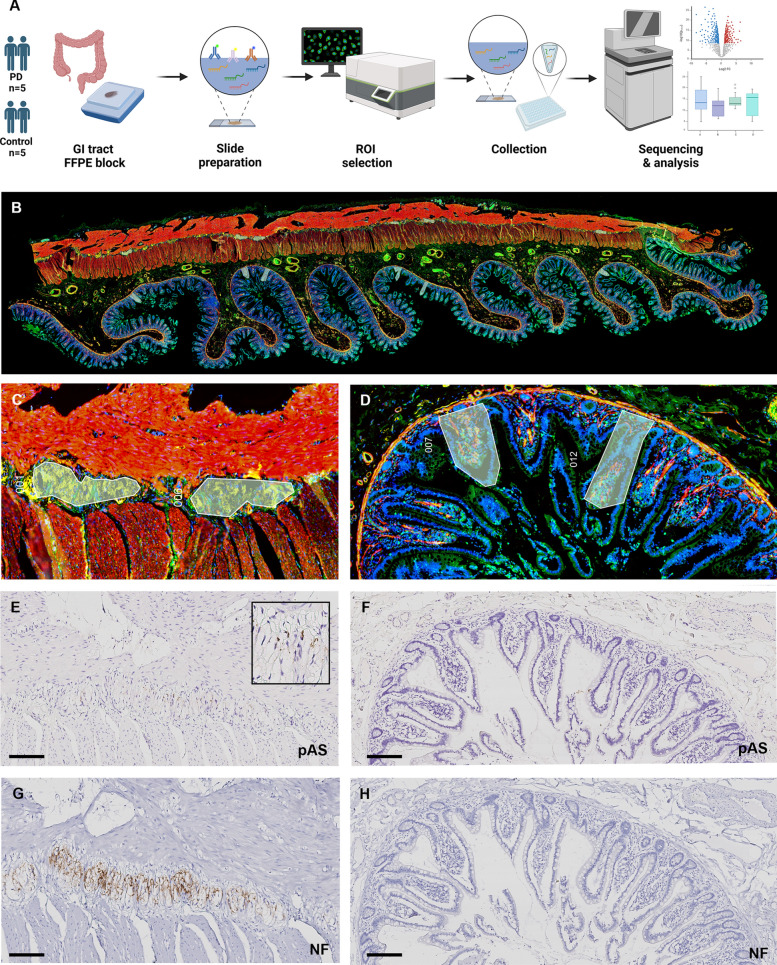
Study process and representative figure of selected ROIs for GeoMx digital spatial profiler of a patient with PD. **A** Study process of the GeoMX digital spatial profiling. **B**–**D** ROI selection of the targeted myenteric plexus (**C**) and intestinal epithelium (**D**). **E**–**H** Representative images of pAS and NF immunostaining of the selected myenteric plexus (**E**), (**G**) and intestinal epithelium (**F**), (**H**). Scale bars: 100 μm. Abbreviations: ROI, region of interest; PD, Parkinson’s disease; pAS, phosphorylated alpha-synuclein; NF, neurofilament

In this study, we utilized the NanoString GeoMx DSP NGS platform along with the Whole Trancriptome Atlas kit to analyze and compare the transcriptomic profiles of patients with PD and controls (Fig. 1). The staining for ROI selection clearly delineated anatomical structures, including the myenteric plexus and intestinal epithelium. Among these, ROIs were carefully selected based on the volume, the presence of pAS staining, and the number of nuclei within each ROI. The nuclei count in ROIs of the myenteric plexus was notably lower (< 100) than that in the intestinal epithelium, which consistently exceeded 100. The area of ROIs of the myenteric plexus were also smaller in size, ranging from 5,000 to 30,000 μm^2^, whereas ROIs of the intestinal epithelium spanned 15,000–150,000 μm^2^. After sequencing, no additional filtering was applied, and all 120 ROIs along with 18,677 gene targets contained in the GeoMx Human Whole Transcriptome Atlas were included in the downstream analysis.

### DEGs between patients with PD and controls

**Fig. 2:**
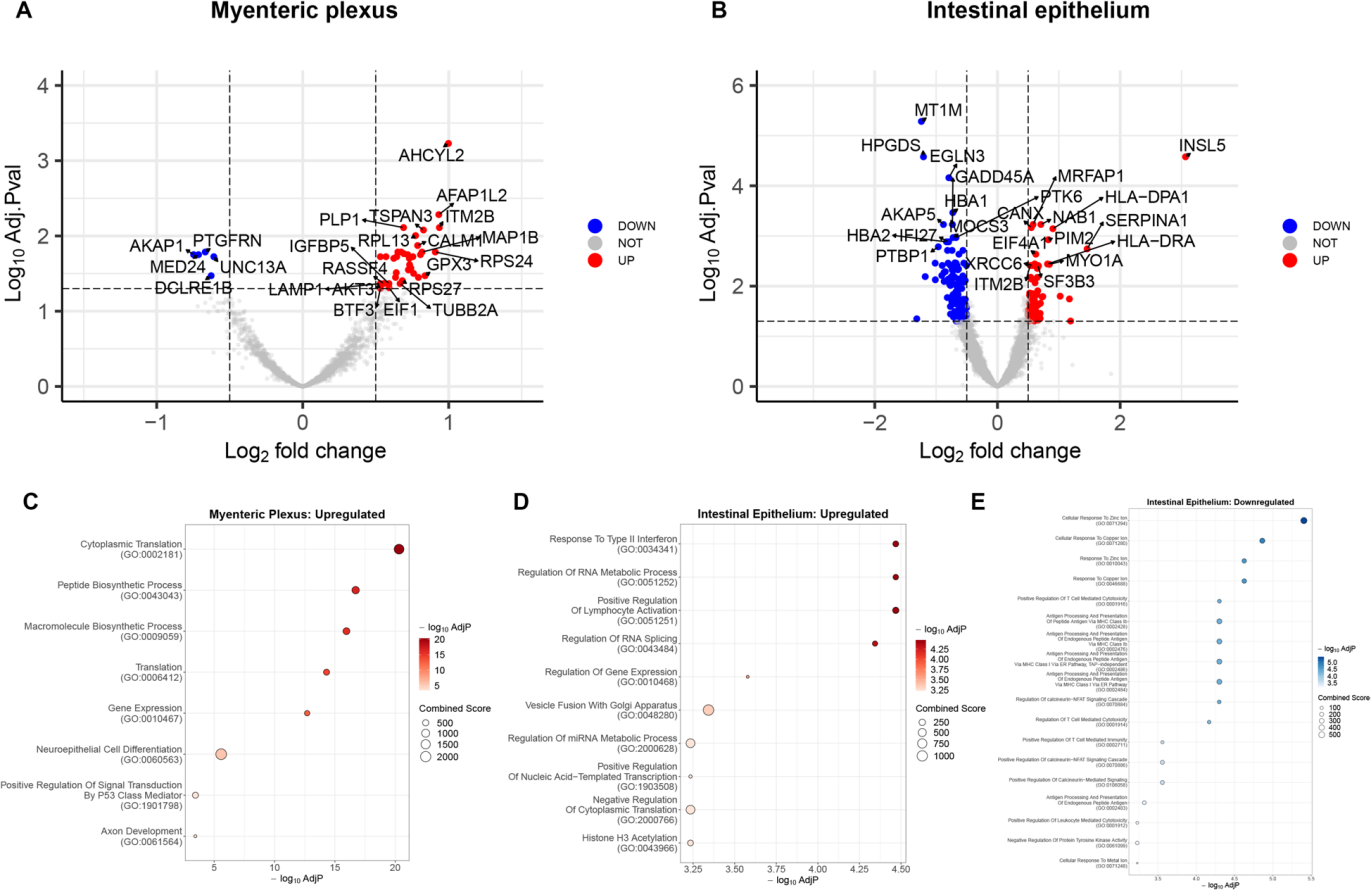
Volcano plots of DEGs and ballon plots of pathway analysis of the myenteric plexus and intestinal epithelium. **A**, **B** Volcano plots. Downregulated and upregulated DEGs (adjusted p-value < 0.05 and absolute Log_2_ fold change > 0.5) in the myenteric plexus (**A**) and intestinal epithelium (**B**) are colored in blue and red, respectively. **C**–**E** Ballon plots of pathway analysis using upregulated and downregulated genes separately in the myenteric plexus (**C**) and intestinal epithelium (**D**), (**E**). Results of pathway analysis of downregulated DEGs in the myenteric plexus are not described because only five downregulated DEGs were found. The full list is available in Supplementary Table 2. Abbreviations: DEG, differentially expressed gene

All DEGs of the myenteric plexus and intestinal epithelium between patients with PD and controls are presented in Table 2. DEG was defined as those with an adjusted *p*-value < 0.05 and an absolute log_2_ fold change greater than 0.5. The distribution of these genes is visualized in the volcano plots shown in Fig. 2A and B. We identified 41 DEGs (36 up-regulated and 5 down-regulated) in the myenteric plexus of patients with PD compared to controls. In the intestinal epithelium, we found 240 DEGs (81 up regulated and 159 down regulated). Due to the substantial number of DEGs identified, we conducted pathway and network analyses to explore their functional impact on the myenteric plexus and intestinal epithelium, as well as the interrelationships among the DEGs.

**Table 2:** DEGs in the myenteric plexus and intestinal epithelium of patients with PD

Location	Direction	DEGs
Myenteric plexus	Upregulated	AFAP1L2, AHCYL2, AKT3, BTF3, C2orf88, CALM1, CD81, CEND1, DDX5, EIF1, GPX3, IGFBP5, ITM2B, LAMP1, MAP1B, NBPF12, PLD3, PLP1, PRMT8, RASSF4, RPL13, RPL18A, RPL27A, RPL35, RPS20, RPS24, RPS27, RPS27A, S100B, SEMA4B, SERPINE2, SPTBN1, SRSF9, TMEM63A, TSPAN3, TUBB2A
	Downregulated	AKAP1, DCLRE1B, MED24, PTGFRN, UNC13A
Intestinal epithelium	Upregulated	ACADVL, AGGF1, AGR2, AKR1B1, ARFGAP3, CANX, CASZ1, CCDC24, CD58, CDHR2, CFB, COL3A1, COL6A1, CPEB2, CPEB4, CYSTM1, DDX5, DERL3, DIAPH2, DNAJA4, DPP8, EIF2A, EIF4A1, GCG, GCH1, GOLGA3, HLA-DPA1, HLA-DRA, HNRNPC, HSD17B4, IGLL5, INSL5, ITM2B, LAMB3, LRRC59, METTL23, MNAT1, MPRIP, MRFAP1, MSI2, MYO1A, NAB1, NBPF12, NEU4, NMD3, NME2, NUB1, PDGFRA, PDIA6, PDLIM7, PES1, PGRMC2, PIM2, PLAT, PQBP1, PRKCSH, PSMB4, RABGGTB, RBM12, RPL22L1, SEC61A1, SERPINA1, SF3B3, SIRPA, SLC25A20, SOCS5, SON, SRP9, SSR4, SUCO, SUPT20H, TFRC, TGFB1, TM9SF4, UBE2Z, UGT2B15, WDR5, XRCC6, YIPF4, YIPF5, ZNF280D
	Downregulated	AKAP5, ALDH1L1, ALDH3B2, ALPL, APOF, AQP8, ATF6, ATP6V0B, ATRX, BAG2, BARHL2, BASP1, BEX4, BTBD3, C10orf95, CA13, CAPN10, CARD14, CCDC51, CD177, CD34, CDC42EP3, CEACAM7, CENPQ, CFAP69, CGB2, CHERP, CLDN10, CMC2, COQ3, CPNE9, CRY1, CRYBB3, CTNND2, DDX4, DDX43, DIS3L, DLAT, DOK5, EBF1, EFR3B, EGLN3, ELF3, ETNK1, FAM133A, FBXO22, FBXW12, FBXW5, FEM1C, FGB, FRMD5, FUOM, GADD45A, GPR12, HBA1, HBA2, HES4, HIC2, HINT2, HLA-A, HLA-B, HOXB5, HPGDS, ICA1L, IFI27, IGSF8, IL23A, KCNJ9, KCNK4, KLK12, KRTAP9-4, LCE3A, LIME1, LIN54, LIX1L, LPIN3, LRWD1, MAPKBP1, MARK3, MAST1, MIA, MICAL2, MICALL2, MICB, MMP13, MOCS3, MT1A, MT1G, MT1M, MT2A, MVB12B, MXI1, MYBL1, MYO7A, NBPF15, NET1, NKX2-4, NOL8, NPY4R, NRXN3, OAF, OBSCN, OR52L1, P4HTM, PCGF2, PDCL3, PEAR1, PHKA2, PHYKPL, PKMYT1, PLAC8, PLEKHG3, PPP2R2B, PRR32, PSMB9, PTBP1, PTK6, RAB19, RAG1, RICTOR, RNF138, RTP5, RUFY2, SARDH, SBK2, SH3D21, SLC13A5, SLC20A2, SLC25A41, SLC26A5, SLC9A3, SPATA2L, SPATC1L, SPPL3, SS18L2, SSTR5, SULT1C3, TAPBPL, TAS2R7, TFAM, THEM5, TIGIT, TINAGL1, TLR1, TMEM102, TMEM41B, TMEM86A, TRAF3IP2, TRIM74, TXK, USP17L12, VPS25, XAGE2, XKR6, YJEFN3, ZC3H8, ZFYVE28, ZNF425, ZNF704

DEG is defined as a gene with an adjusted *p*-value < 0.05

DEG, differentially expressed gene; PD, Parkinson’s disease

### Pathway analysis results

The significant findings from the pathway analysis are summarized in Table 3 and Fig. 2C–E. As only five DEGs were downregulated in the myenteric plexus, their pathway analysis results were excluded from Table 3 and Fig. 2C–E (a full list of results is available in Supplementary Table 1). In Fig. 2C–E, pathways positioned higher on the plot indicate greater statistical significance, while larger circle sizes represent higher combined scores, further reflecting the relevance of the corresponding pathways. Upregulated and downregulated pathways are indicated in red and blue, respectively.

**Table 3:** Results of pathway analysis of DEGs in patients with PD

Location	Direction	Term	Overlap	*p*-value	Adjusted p-value	Odds Ratio	Combined Score	Genes
Myenteric plexus*	Upregulated	Cytoplasmic Translation (GO:0002181)	8/93	4.37021E-12	1.50772E-09	66.8	1747.8	RPS27;RPL18A;RPL27A;RPL35;RPL13;RPS20;RPS27A;RPS24
		Peptide Biosynthetic Process (GO:0043043)	8/158	3.18316E-10	5.49096E-08	37.7	825.3	RPS27;RPL18A;RPL27A;RPL35;RPL13;RPS20;RPS27A;RPS24
		Macromolecule Biosynthetic Process (GO:0009059)	8/183	1.02439E-09	1.17805E-07	32.3	668.8	RPS27;RPL18A;RPL27A;RPL35;RPL13;RPS20;RPS27A;RPS24
		Translation (GO:0006412)	8/234	7.10712E-09	6.12989E-07	25.0	468.2	RPS27;RPL18A;RPL27A;RPL35;RPL13;RPS20;RPS27A;RPS24
		Gene Expression (GO:0010467)	8/296	4.422E-08	3.05118E-06	19.5	330.6	RPS27;RPL18A;RPL27A;RPL35;RPL13;RPS20;RPS27A;RPS24
		Neuroepithelial Cell Differentiation (GO:0060563)	2/7	0.0001	0.0038	234.8	2261.1	TUBB2A;MAP1B
		Positive Regulation Of Signal Transduction By P53 Class Mediator (GO:1,901,798)	2/21	0.0006	0.0319	61.7	453.4	DDX5;RPS20
		Axon Development (GO:0061564)	3/99	0.0007	0.0322	18.8	135.5	MAP1B;PLP1;S100B
Intestinal epithelium	Upregulated	Response To Type II Interferon (GO:0034341)	5/80	1.8223E-05	0.0115	17.4	190.0	NUB1;GCH1;SIRPA;CD58;HLA-DPA1
		Regulation Of RNA Metabolic Process (GO:0051252)	5/91	3.40478E-05	0.0115	15.2	156.1	SF3B3;SON;SUPT20H;RBM12;PQBP1
		Positive Regulation Of Lymphocyte Activation (GO:0051251)	4/47	3.90017E-05	0.0115	24.0	243.8	XRCC6;SIRPA;HLA-DRA;HLA-DPA1
		Regulation Of RNA Splicing (GO:0043484)	5/102	5.88997E-05	0.0130	13.4	130.9	SF3B3;SON;SUPT20H;RBM12;PQBP1
		Regulation Of Gene Expression (GO:0010468)	14/1127	0.0002	0.0279	3.5	30.9	SF3B3;TGFB1;TFRC;NAB1;AKR1B1;PQBP1;ZNF280D;SON;DNAJA4;WDR5;AGR2;SIRPA;SUPT20H;RBM12
		Vesicle Fusion With Golgi Apparatus (GO:0048280)	2/6	0.0002	0.0353	126.0	1050.3	YIPF4;YIPF5
		Positive Regulation Of Nucleic Acid-Templated Transcription (GO:1,903,508)	9/557	0.0004	0.0394	4.4	34.4	CASZ1;XRCC6;SF3B3;TGFB1;WDR5;NME2;AKR1B1;PIM2;SUPT20H
		Histone H3 Acetylation (GO:0043966)	3/36	0.0004	0.0394	23.2	180.5	SF3B3;WDR5;SUPT20H
		Regulation Of miRNA Metabolic Process (GO:2,000,628)	2/8	0.0004	0.0394	84.0	648.1	DDX5;TGFB1
		Negative Regulation Of Cytoplasmic Translation (GO:2,000,766)	2/8	0.0004	0.0394	84.0	648.1	CPEB2;CPEB4
	Downregulated	Cellular Response To Zinc Ion (GO:0071294)	4/15	4.90349E-06	0.0045	46.5	568.8	MT2A;MT1A;MT1M;MT1G
		Cellular Response To Copper Ion (GO:0071280)	4/20	1.68734E-05	0.0077	32.0	351.4	MT2A;MT1A;MT1M;MT1G
		Response To Copper Ion (GO:0046688)	4/24	3.6101E-05	0.0098	25.6	261.6	MT2A;MT1A;MT1M;MT1G
		Response To Zinc Ion (GO:0010043)	4/25	4.27121E-05	0.0098	24.4	245.1	MT2A;MT1A;MT1M;MT1G
		Positive Regulation Of T Cell Mediated Cytotoxicity (GO:0001916)	4/33	0.0001	0.0136	17.6	157.6	IL23A;HLA-B;HLA-A;MICB
		Antigen Processing And Presentation Of Endogenous Peptide Antigen Via MHC Class I Via ER Pathway (GO:0002484)	3/13	0.0001	0.0136	38.1	340.4	HLA-B;HLA-A;MICB
		Antigen Processing And Presentation Of Endogenous Peptide Antigen Via MHC Class I Via ER Pathway, TAP-independent (GO:0002486)	3/13	0.0001	0.0136	38.1	340.4	HLA-B;HLA-A;MICB
		Antigen Processing And Presentation Of Endogenous Peptide Antigen Via MHC Class Ib (GO:0002476)	3/13	0.0001	0.0136	38.1	340.4	HLA-B;HLA-A;MICB
		Antigen Processing And Presentation Of Peptide Antigen Via MHC Class Ib (GO:0002428)	3/13	0.0001	0.0136	38.1	340.4	HLA-B;HLA-A;MICB
		Regulation Of calcineurin-NFAT Signaling Cascade (GO:0070884)	4/34	0.0001	0.0136	17.0	150.3	PTBP1;AKAP5;SPPL3;CHERP
		Regulation Of T Cell Mediated Cytotoxicity (GO:0001914)	4/36	0.0002	0.0155	16.0	137.2	IL23A;HLA-B;HLA-A;MICB
		Positive Regulation Of T Cell Mediated Immunity (GO:0002711)	4/43	0.0004	0.0285	13.1	103.4	IL23A;HLA-B;HLA-A;MICB
		Positive Regulation Of calcineurin-NFAT Signaling Cascade (GO:0070886)	3/19	0.0004	0.0285	23.8	184.4	PTBP1;SPPL3;CHERP
		Positive Regulation Of Calcineurin-Mediated Signaling (GO:0106058)	3/19	0.0004	0.0285	23.8	184.4	PTBP1;SPPL3;CHERP
		Antigen Processing And Presentation Of Endogenous Peptide Antigen (GO:0002483)	3/21	0.0006	0.0361	21.2	157.5	HLA-B;HLA-A;MICB
		Positive Regulation Of Leukocyte Mediated Cytotoxicity (GO:0001912)	4/51	0.0007	0.0396	10.9	78.7	IL23A;HLA-B;HLA-A;MICB
		Cellular Response To Metal Ion (GO:0071248)	6/135	0.0007	0.0396	6.0	43.2	CPNE9;MT2A;MT1A;MT1M;MT1G;SLC13A5
		Negative Regulation Of Protein Tyrosine Kinase Activity (GO:0061099)	3/23	0.0008	0.0396	19.1	136.5	ZFYVE28;PTK6;VPS25

Pathways with adjusted *p*-value < 0.05 are listed. GO Biological Process 2023 is used for interpretation of pathway analysis

^*^Results of pathway analysis of downregulated DEGs in the myenteric plexus are not described because only five downregulated DEGs were found. The full list is available in Table S2

DEG, differentially expressed gene; PD, Parkinson’s disease

In the myenteric plexus, upregulated pathways such as neuroepithelial cell differentiation, associated with the *TUBB2A* and *MAP1B*, and axon development, linked to *MAP1B*, *PLP1*, and *S100B*, were noteworthy in the GO Biological Process 2023. In the intestinal epithelium, pathways related to the response to type II interferon and positive regulation of lymphocyte activation were upregulated, while pathways associated with cellular response to zinc and copper ions, positive regulation of T-cell-mediated cytotoxicity, and antigen processing and presentation of endogenous peptide antigen were downregulated.

### Network analysis results

**Fig. 3:**
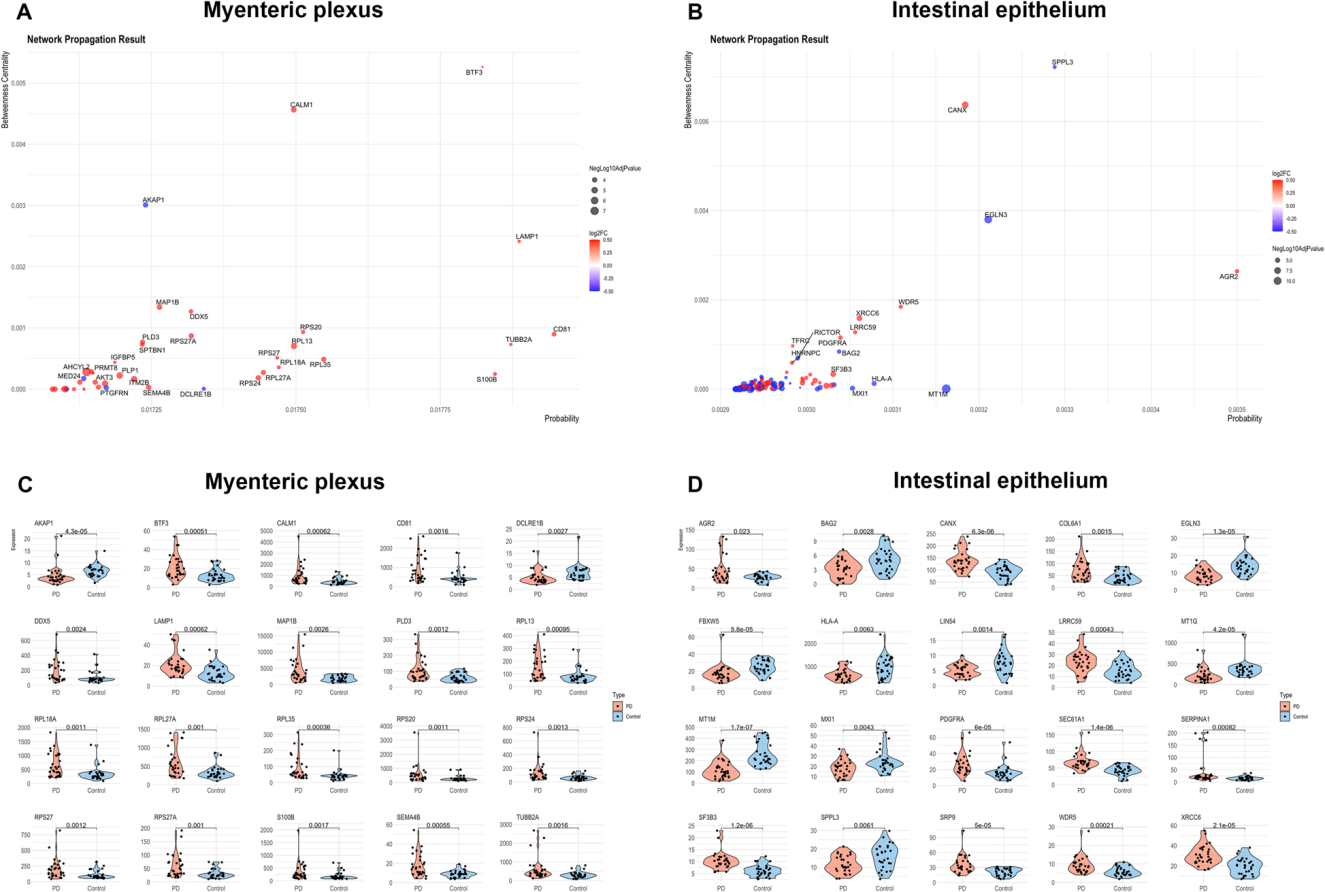
Network analysis and included top 20 DEGs in the myenteric plexus and intestinal epithelium. **A**, **B** Dot plots displaying network analysis results of DEGs in the myenteric plexus (**A**) and intestinal epithelium (**B**). The y-axis represents the betweenness centrality and the x-axis represents the probability calculated using random work with restart algorithm. **C**, **D** Dot and violin plots of top 20 DEGs with the highest probabilities from the network analysis of the myenteric plexus (**C**) and intestinal epithelium (**D**). Each dot corresponds to the outcome of the bulk spatial transcriptomic analysis performed on the respective targeted region of interest. Abbreviations: DEG, differentially expressed gene

The results of the network analysis for identifying key DEGs in both the myenteric plexus and intestinal epithelium are summarized in Fig. 3A, B, and Supplementary Table 2. In Fig. 3A and B, the x-axis represents the probability of each gene, reflecting its statistical significance, while the y-axis denotes betweenness centrality, indicating the extent to which the gene functions as a hub interacting with other genes. Therefore, genes located in the upper right quadrant of the plot are considered more significant in the network analysis. Red dots indicate upregulated genes, while blue dots represent downregulated genes. Among these, the 20 genes with the highest probability were selected to directly compare expression differences between patients with PD and controls, as visualized in the dot and violin plots (Fig. 3C and D).

Although these genes are likely to be key nodes in the network analysis, we further refined our selection by identifying those that also showed significant impact in the pathway analysis among the top 20 genes to highlight potentially more critical genes. Finally, several key genes were identified as follows: In the myenteric plexus, these included *TUBB2A*, *MAP1B*, *DDX5*, *S100B*, and ribosomal genes such as *RPL13*, *RPL18A*, *RPL27A*, *RPL35*, *RPS20*, *RPS24*, and *RPS27A*. In the intestinal epithelium, key genes included *AGR2*, *BAG2*, *HLA-A*, *MT1G*, *MT1M*, *SF3B3*, *SPPL3*, *WDR5*, and *XRCC6*.

## Discussion

This was the first study to identify changes in DEGs in the myenteric plexus and intestinal epithelium of patients with PD of which AS accumulation was confirmed in the myenteric plexus. The pathway alterations in the intestinal epithelium are primarily related to inflammatory processes. Specifically, response to type II interferon (GO:0034341) and positive regulation of lymphocyte activation (GO:0051251) were upregulated, while positive regulation of T cell-mediated cytotoxicity (GO:0001916) was downregulated. In the upregulated pathways, *HLA-DRA* and *HLA-DPA1* encode alpha chain paralogues of the major histocompatibility complex (MHC) class II. These proteins are expressed on antigen-presenting cells and play a crucial role in activating the adaptive immune system via CD4⁺ T cells [14]. In the downregulated pathways, *HLA-A* and *HLA-B* encode transmembrane components of MHC class I and are critical for CD8⁺ T cell-mediated cytotoxicity [15]. These findings are in line with those reported in a recent study by Bolen et al., which employed single-cell multiomics analysis of colon biopsies from patients with PD [16]. In that study, genes related to cell adhesion, T cell migration, and myeloid differentiation were depleted in colonocytes in patients with PD, whereas gene expression associated with T cell activation and responses to external stimuli was upregulated. Although Bolen et al. did not report the specific genes contributing to each pathway, these results implicate gene expression alterations regarding both intrinsic and adaptive immune systems in the intestinal epithelium of patients with PD.

Previous studies have suggested the presence of inflammatory changes in the intestinal epithelium of patients with PD indirectly by evaluating alterations in microbiome composition [17]. Increased abundance of the genera *Akkermansia* and *Bifidobacterium,* and decreased abundance of the genera *Roseburia* and *Faecalibacterium* in the GI tract have been reported in patient with PD [18]. Additionally, fecal short-chain fatty acids, fecal inflammatory markers such as CXCL18 and IL-1β, and zonulin, the intestinal tight junction protein, have been associated not only with microbial composition and diversity but also with the onset of motor symptoms, suggesting a potential link to development of PD [19]. Microbial alterations, disruption of intestinal defense mechanisms, and dysfunction of the tight junction contribute to pro-inflammatory conditions in the intestinal epithelium. Moreover, key genes found in this study also suggested the inflammatory changes in the intestinal epithelium. *SERPINA1*, a gene belonging to the serpin superfamily, is an acute-phase protein with autophagy-related functions, which serves as a marker of the overall immune response. A *SERPINA1* isoform analysis in cerebrospinal fluid revealed that the most acidic isoform was significantly elevated in patients with PD dementia [20], and *SERPINA1* protein levels were upregulated in the plasma of patients with PD [21]. Metallothioneins (*MT1* and *MT2*) were downregulated genes involved in cellular response to zinc ion (GO:0071294) and cellular response to copper ion (GO:0071280) in this study. These are known to have anti-inflammatory functions and are also related to intestinal inflammation [22]. Therefore, the findings of this study imply that environmental pro-inflammatory changes may contribute to alterations in immune-related gene expression within the intestinal epithelium in PD.

The myenteric plexus plays a crucial role in coordinating muscle movements for peristalsis of the GI tract, where AS accumulation is most commonly observed in patients with PD [23]. Pathway analysis of the myenteric plexus showed upregulation of neuronal regeneration pathways such as neuroepithelial cell differentiation (GO:0060563) and axon development (GO:0061564). Neuroepithelial cells function as neural stem cells, which are capable of renewing themselves and generating neurons and glial cells after differentiation [24]. Approximately 1% of isolated gut cells in the GI tract of both humans and animals function as stem cells, possessing self-renewing capacity [25]. Among the identified key genes, *TUBB2A* encodes a neuronal-specific isotype of beta-tubulin, which is frequently found in the myenteric plexus. *TUBB2A* plays a crucial role in mitosis, intracellular transport, and cortical development, including neuronal proliferation, migration and cortical laminar organization [26]. Although no studies have directly investigated the implications of *TUBB2A* upregulation in the myenteric plexus, its known biological function implicates a potential increase in neuronal development and differentiation.

*LAMP1*, one of the upregulated genes with the highest probability in the network analysis, encodes a membrane glycoprotein localized to lysosomes and endosomes. *LAMP1* primarily functions in the autophagy pathway and has been shown to mitigate AS toxicity in a Drosophila model [27]. Moreover, *LAMP1* upregulation reduced pathological AS accumulation and protected dopaminergic neurons in cellular models [28]. *S100B* is a calcium-binding protein, which is predominantly expressed in astrocytes in the brain and in enteric glial cells [29]. *S100B* interacts with the receptor for advanced glycation end products, leading to the activation and migration of microglia, thereby promoting a pro-inflammatory condition [29, 30]. Studies have demonstrated that *S100B* protein is directly involved in gut inflammatory disease such as Crohn’s disease and ulcerative colitis [29, 31]. There is no direct evidence which reported *S100B* elevation in the myenteric plexus of PD, but a post-mortem study found elevated expression of *S100B* in the substantia nigra of patients with PD [32]. Previous studies using animal models have suggested an association between gut inflammation and AS accumulation in the enteric nervous system. Elevated pAS levels and dopaminergic neuronal loss have been observed in both the brain and colon of PD monkey models intoxicated with 1-methyl-4-phenyl-1,2,3,6-tetrahydropyridine (MPTP) [33]. Moreover, inflammatory changes in the colon are known to enhance AS expression and phosphorylation in the myenteric plexus, as demonstrated in animal studies [34]. Additionally, enteroendocrine cells, which are components of the intestinal epithelium, are directly connected to enteric neurons. It has been reported that AS accumulation in these cells can be transmitted to enteric nerves, which are subsequently connected to the myenteric plexus [35]. Therefore, this study demonstrates that intestinal inflammation, AS accumulation, and neuronal protection in the myenteric plexus may occur concurrently in the colon of patients with PD and provides a basis for future investigations into the potential protective effects of stem cell-driven regeneration in the myenteric plexus of patients with PD.

### Limitations

This study has several limitations that should be considered when interpreting the results. First, as this is a retrospective study, the clinical status of PD of the patients at the time of surgery could not be evaluated. Consequently, we were unable to determine the clinical relevance of gene alterations in this study. Second, the interpretation of the gut-brain axis pathophysiology and the significance of AS accumulation in relation to the gene alterations in the myenteric plexus is limited due to the cross-sectional nature of this study. Finally, one of the patients with PD had a history of metastatic cancer and hepatectomy, which may have influenced the results. However, because the metastasis was confined to the liver and no regional lymph node was involved, there would be no transcriptomic alterations in the normal colonic tissue of the patient.

## Conclusions

Our study demonstrated that simultaneous alterations of gene expression profiles occur in both the myenteric plexus and intestinal epithelium of patients with PD. Notably, an inflammatory processes may occur in the intestinal epithelium, while neuronal regeneration mechanisms may be active in the myenteric plexus in patients with overtly developed PD. Future studies are warranted to elucidate their roles in the pathophysiology of PD and potential association between AS accumulation in the myenteric plexus of the colon and significantly identified genes such as *LAMP1*, *TUBB2A*, and *S100B* in the myenteric plexus, and *HLA-DRA*, *SERPINA1*, and metallothioneins in the intestinal epithelium. A spatial transcriptomic analysis of the brain and GI tract will provide a better understanding of the gut-brain axis in PD. 

## Supplementary Information

Additional file 1.

Additional file 2.

## Data Availability

All data are available in the main text or Supplementary Materials. Any requests to access the raw data and pathological slides will be considered by the corresponding author Professor Han-Joon Kim (movement@snu.ac.kr). When a decision about the request is made, fully anonymized data will be provided under permission of the Institutional Review Board of the Seoul National University Hospital.

## Abbreviations

PD: Parkinson’s disease
AS: Alpha-synuclein
pAS: Phosphorylated alpha-synuclein
GI: Gastrointestinal
SNUH: Seoul National University Hospital
FFPE: Formalin-fixed paraffin-embedded
IHC: Immunohistochemistry
NF: Neurofilament
DSP: Digital Spatial Profiler
DEPC: Diethylpyrocarbonate
SSC: Saline-sodium citrate
PGP 9.5: Protein gene product 9.5
α-SMA: α-Smooth muscle actin
ROI: Region of interest
DCC: Digital Count Conversion
QC: Initial quality control
DEG: Differentially expressed gene
PPI: Protein–protein interaction
RWR: Random walk with restart
MHC: Major histocompatibility complex
